# Quantitative multiparametric MRI allows safe surgical planning in patients undergoing liver resection for colorectal liver metastases: report of two patients

**DOI:** 10.1259/bjrcr.20200172

**Published:** 2021-01-12

**Authors:** Pulkit Sethi, Navamayooran Thavanesan, Fenella KS Welsh, John Connell, Elisabeth Pickles, Matt Kelly, Jonathan A Fallowfield, Timothy J Kendall, Damian J Mole, Myrddin Rees

**Affiliations:** 1 Department of Hepatobiliary Surgery, Basingstoke and North Hampshire Hospital, Basingstoke, Hampshire, United Kingdom; 2 Perspectum, Gemini One, Oxford, United Kingdom; 3 Institute of Biomedical Engineering, University of Oxford, Oxford, UK; 4 Centre for Inflammation Research, Queen’s Medical Research Institute, University of Edinburgh, Edinburgh, UK; 5 Clinical Surgery, University of Edinburgh, Edinburgh, UK

## Abstract

It is not uncommon for clinicians to encounter varying degrees of hepatic steatosis in patients undergoing resection for colorectal liver metastases (CRLM). Magnetic resonance imaging is currently the preferred investigation for identification and pre-operative planning of these patients. An objective assessment of liver quality and degree of steatosis is paramount for planning a safe resection, which is seldom provided by routine MRI sequences.

We studied two patients who underwent an additional pre-operative multiparametric MRI scan (LiverMultiScan^TM^) as a part of an observational clinical trial (HepaT1ca, NCT03213314) to assess the quality of liver. Outcome was assessed in the form of post-hepatectomy liver failure.

Both patients (Patient 1 and 2) had comparable pre-operative characteristics. Both patients were planned for an extended right hepatectomy with an estimated future liver remnant of approximately 30%. Conventional preoperative contrast MRI showed mild liver steatosis in both patients. Patient one developed post-hepatectomy liver failure leading to prolonged hospital stay compared to patient two who had uneventful post-operative course. Retrospective evaluation of multiparametric MRI scan revealed findings consistent with fibro-inflammatory disease and steatosis (cT1 829 ms, PDFF 14%) for patient 1 whereas patient two had normal parameters (cT1 735 ms, PDFF 2.4%). These findings corresponded with the resection specimen histology.

Multiparametric MRI can objectively evaluate future liver health and volume which may help refine surgical decision-making and improve patient outcomes

## Introduction

Safer liver surgery requires a holistic approach to pre-operative assessment of the risk of post-hepatectomy liver dysfunction. Advances in systemic anti cancer therapy for colorectal liver metastases prior to surgical resection have resulted in significant gains, with partial responses seen in up to 50% of patients,^1^ allowing more individuals to be eligible for curative resection. However, pre-existing liver disease (typically undiagnosed) and chemotherapy-associated hepatotoxicity can strongly influence surgical outcomes, highlighting the need for careful pre-operative evaluation. Indeed, the incidence of post-hepatectomy liver failure (PHLF) in individuals undergoing surgery may exceed 20% in patients with chronic liver disease.^2^ Contrast-enhanced liver MRI is now the preferred modality for detecting liver metastases and may also be used for pre-operative volumetric evaluation. Liver health can be assessed using imaging tests, liver biopsy or specialised assays, for example indocyanine green clearance, but no approach by far, has been able to objectively plan a safe resection, within permissible FLR limits.^3–6^ Accurate information about future liver performance is paramount in planning for safe liver resection, which depends upon both the quality and volume of the future functional liver remnant (FLR) to avoid PHLF.^2,7–9^ Although histological assessment by liver biopsy is considered the gold standard for diagnosing liver disease, it is not performed routinely due to its invasive nature and the potential risk of complications such as bleeding and bile leak.

T1 relaxation time in the liver can be used as a biomarker for fibrosis and inflammation.^10^ As fibrosis and inflammation increase, so does the amount of extracellular water in the liver, leading to an increase in T1 relaxation time. However, the magnetic properties of hepatic iron lowers the T1 measurement which, if unaccounted for, would result in an underestimate of the true T1 relaxation time. Iron-corrected T1 is achieved by measuring T2* relaxation time, from which iron concentration can be calculated. This can then be used to adjust the T1 measurement, to give cT1 (iron-corrected T1 relaxation time). Liver*MultiScan*^TM^ is a quantitative multiparametric MRI technology with high diagnostic accuracy for hepatic fibro-inflammation, steatosis and iron content,^11,12^ as well as predicting liver-related outcomes in patients with chronic liver disease.^13^

This technology has been developed into, Hepatica^TM^, that combines semi-automatic calculation of FLR volume with cT1 to predict future liver performance (FLP). In a recently conducted study on 77 patients, where *≥* 10% of liver volume was resected, the median length of hospital stay was found to be longer for those with a preoperative cT1 above the upper limit of normal compared to those with cT1 within the normal range.^14^

Briefly, the quantitative cT1 data are collected using five axial 8 mm thick slices of T1 maps (modified Look-Locker inversion recovery) with 20 mm centre-to-centre spacing across the liver centred on the porta hepatis; the T1-shortening effect of hepatic iron is then corrected for by measuring T2* (fitting of the in-phase multiecho gradient echo images). Liver fat is measured using proton-density fat fraction mapping (PDFF) of five axial 10 mm thick slices of multiecho gradient echo images analysed using the iterative decomposition of water and fat with echo asymmetry and least squares estimation method (IDEAL).^15^ Full images are typically acquired in 5–6 min with no use of contrast agents and are analysed centrally and returned to the physician.

Here, we present two individual clinical experiences that illustrate the potential utility of using augmented non-invasive MRI in the pre-operative evaluation of patients undergoing liver resection for colorectal metastases.

## Clinical presentation

Two patients with similar disease presentation were studied retrospectively. The demographics, preoperative (Table 1) and post-operative patient characteristics were compared.

**Table 1.** Patient characteristics and baseline laboratory tests 2 weeks prior to surgery

	**Patient 1**	**Patient 2**
Age	51	58
BMI (kg/m^2^)	33.1	29.7
ECOG score	0	0
Known liver disease	None	None
Other comorbidities	None	Hypertension
Alcohol intake	1–10 units/week	1–10 units/week
Concomitant medications	None	Ramipril, Amlodipine, Bendroflumethazide, Mirtazapine, Citalopram, Ranitidine
**Baseline laboratory value (normal reference values**)		
Platelets (10^9^/L) (150-400)	228	118
INR (0.9–1.2)	0.9	1.0
Bilirubin (µmol/L)^3–20^	10	10
Albumin (g/L) (33-49)	41	35
AST (U/L) (5-30)	35	30
ALT (U/L) (5-30)	30	21
ALP (U/L) (50-100)	91	119
GGT (U/L) (6-50)	35	120
**Operative characteristics**		
Operation	Extended Right Hepatectomy	Extended Right Hepatectomy
Duration of surgery (min)	210	150
Intermittent clamp time (min)	21	19
Blood loss (ml)	450	270

ALP, alkaline phosphatase; ALT, alanine transaminase; AST, aspartate transaminase; BMI, body mass index;ECOG, Eastern Cooperative Oncology Group;GGT, gamma-glutamyl transpeptidase; INR, international normalised ratio.

### Patient 1

A 51-year-old male who had been surgically treated for a colorectal primary two years ago, presented with metachronous right-sided liver metastases. He underwent downstaging neoadjuvant chemotherapy with 14 cycles of FOLFOX (folinic acid, 5-fluorouracil and oxaliplatin) over the course of four months, resulting in a partial response. Conventional liver MRI showed mild steatosis and an estimated FLR of approximately 30%. After a 6-week break from chemotherapy, the patient was re-assessed and a plan for extended right hepatectomy was made (segments 5,6,7 & eight with extension into 4B, sparing the middle hepatic vein). At surgery, the liver appeared fatty and the procedure was completed in 3.5 h with a Pringle clamp time of 21 min and 450 ml blood loss. There was no requirement for red cell transfusion.

**Figure 1.**
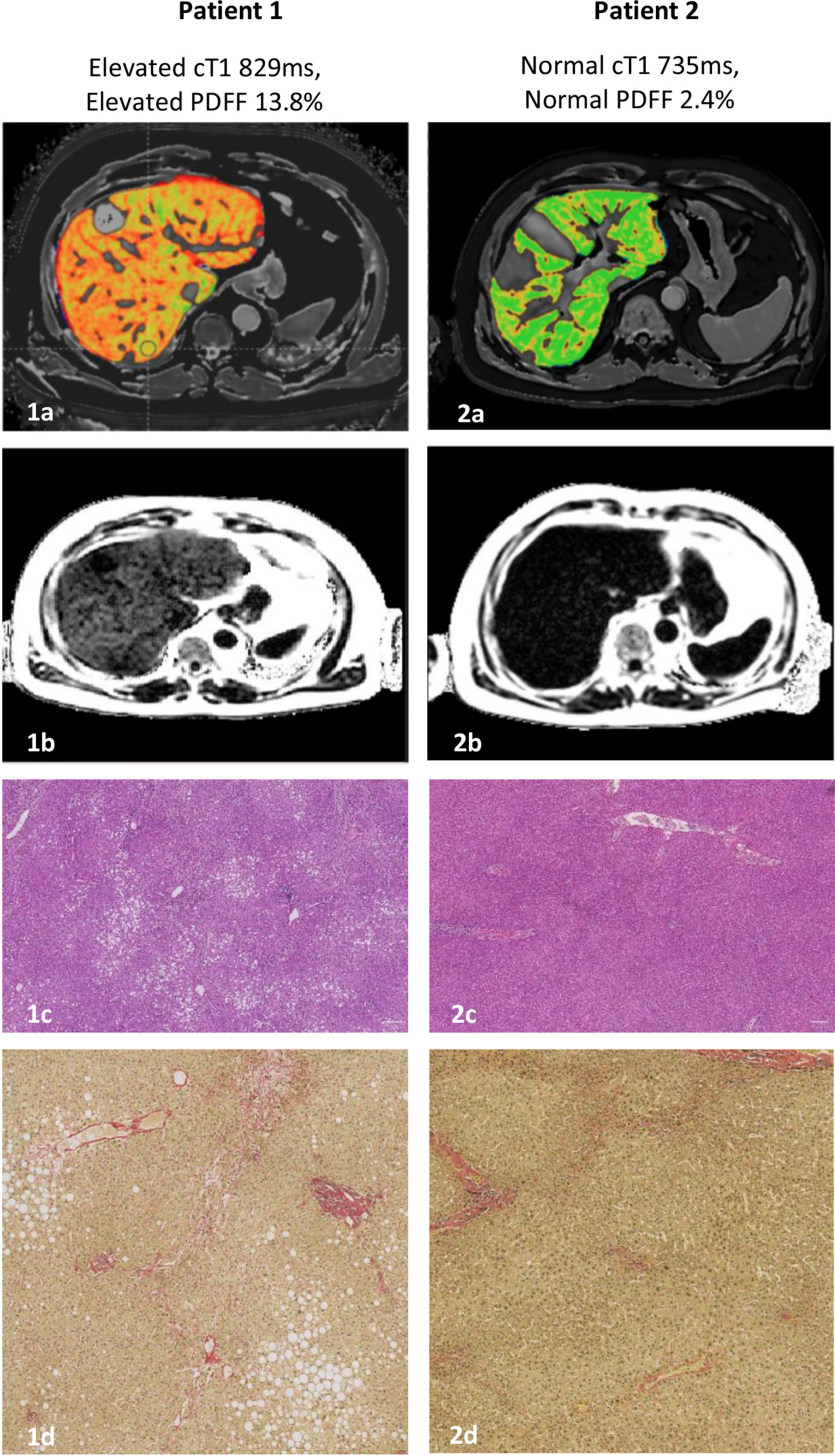


Post-operatively, the patient developed liver failure complicated by pleural effusion, infected ascites and paralytic ileus. The patient underwent intensive management with antibiotics, human albumin transfusions, diuretics and image-guided drainage of the effusion. He was discharged on post-operative day 15, this being substantially longer than the median hospital stay of 4 days in our unit. Histological assessment of the resected background liver parenchyma showed Grade two steatosis on the Kleiner-Brunt scale, 1.62% corrected steatosis by pixel classification of whole-slide image with peri-central sinusoidal dilatation and accompanying sinusoidal fibrosis, 2.75% corrected fibrosis by pixel classification^16^ of whole-slide image (Figure 1: 2a,2b).

### Patient 2

A 58-year-old male presented with metachronous right-sided colorectal metastases received six cycles of neoadjuvant FOLFOX with panitumumab, stopping treatment 8 weeks prior to liver resection following a partial response. Conventional liver MRI showed minimal steatosis with an estimated FLR of approximately 30%. He underwent a right hepatectomy with extension into segment 4B, sparing the middle hepatic vein. Surgery was completed in 2.5 h with a clamp time of 19 min and 270 ml of blood loss and the patient did not require red cell transfusion. Notably, the liver appeared healthy during the surgery.

The post-operative course was uncomplicated and the patient was discharged on day three following surgery. The resected background liver parenchyma showed no histological evidence of fat inflammation, ballooning, fibrosis or sinusoidal dilatation (Figure 1: 2c,2d).

These patients had been enrolled in an observational clinical trial (HepaT1ca, NCT03213314)^17^ and additional imaging using multiparametric liver MRI (LiverMultiScan^TM^, Perspectum Diagnostics, Oxford, UK) was performed as part of the study, with the care team blinded to the results. Liver steatosis was graded histologically according to the Kleiner-Brunt scale.^18^ Outcomes were studied in the form of hospital stay and post-hepatectomy liver failure, defined as per the International Study Group of Liver Surgery (ISGLS) criteria.^19^

## Multiparametric liver MRI findings

For Patient 1, the FLR was calculated as 23% and the iron-corrected T1 relaxation time (cT1) of the liver parenchyma measured 829 ms (normal <795 ms), indicating undiagnosed fibro-inflammatory disease while the MRI proton-density fat fraction (PDFF)^11–13,20^ of 14% (normal <5%) indicated steatosis (Figure 1:1a,1b). In contrast, Patient 2 was calculated to have a FLR of 29% with a cT1 of 735 ms and PDFF of 2.4%, which are both within the normal range (Figure 1**:**2a, 2b).

## Discussion

This report describes the valuable information offered by pre-operative quantitative multiparametric MRI used in two individuals who presented seemingly similar surgical challenges at the outset, yet who had very different post-operative outcomes. The additional imaging revealed significant unsuspected liver disease in Patient 1. Identifying this pre-operatively could have changed the surgical decision-making to a two-staged procedure (portal vein embolization/ligation followed by right hepatectomy) which in turn might have averted post-operative liver failure and his delayed recovery.

Conversely, the additional imaging in Patient 2 confirmed healthy parenchymal liver tissue characteristics. This information would have justified the plan to proceed with a single-staged procedure.

It is widely understood that chemotherapy-associated steatohepatitis is an unpredictable phenomenon that occurs in some patients but not others. It is most commonly diagnosed histologically after surgery has taken place. These two patient’s experiences highlight the significant potential of utilising pre-operative quantitative multiparametric MRI to accurately evaluate liver tissue health to help refine surgical decision-making and improve patient outcomes.

## Learning points

- Multiparametric MRI can help in analysis of liver tissue composition.
- Multiparametric MRI can objectively evaluate future liver health and volume, which may help refine surgical decision making.
- It may be possible to predict the outcomes of patients.

## References

[1] Swan PJ, Welsh FKS, Chandrakumaran K, Rees M. Long-Term survival following delayed presentation and resection of colorectal liver metastases. Br J Surg 2011; 98: 1309–17. doi: 10.1002/bjs.7527 21598236

[2] Khan AS, Garcia-Aroz S, Ansari MA, Atiq SM, Senter-Zapata M, Fowler K, et al. Assessment and optimization of liver volume before major hepatic resection: current guidelines and a narrative review. Int J Surg 2018; 52: 74–81. doi: 10.1016/j.ijsu.2018.01.042 29425829

[3] Zorzi D, Laurent A, Pawlik TM, Lauwers GY, Vauthey J-N, Abdalla EK. Chemotherapy-Associated hepatotoxicity and surgery for colorectal liver metastases. Br J Surg 2007; 94: 274–86. doi: 10.1002/bjs.5719 17315288

[4] Robinson SM, Wilson CH, Burt AD, Manas DM, White SA. Chemotherapy-Associated liver injury in patients with colorectal liver metastases: a systematic review and meta-analysis. Ann Surg Oncol 2012; 19: 4287–99. doi: 10.1245/s10434-012-2438-8 22766981 PMC3505531

[5] Zhang YN, Fowler KJ, Hamilton G, Cui JY, Sy EZ, Balanay M, et al. Liver fat imaging-a clinical overview of ultrasound, CT, and MR imaging. Br J Radiol 2018; 91: 20170959. doi: 10.1259/bjr.20170959 29722568 PMC6223150

[6] Gómez-Ramírez J, Martín-Pérez E, Amat CG, Sanz IG, Bermejo E, Rodríguez A, et al. Influence of pre-surgical chemotherapy on liver parenchyma and post-surgical outcome of patients subjected to hepatectomy due to colorectal carcinoma metastases. Cir Esp 2010; 88: 404–12. doi: 10.1016/S2173-5077(10)70047-8 20971458

[7] Tamandl D, Klinger M, Eipeldauer S, Herberger B, Kaczirek K, Gruenberger B, et al. Sinusoidal obstruction syndrome impairs long-term outcome of colorectal liver metastases treated with resection after neoadjuvant chemotherapy. Ann Surg Oncol 2011; 18: 421–30. doi: 10.1245/s10434-010-1317-4 20844968

[8] Chan G, Hassanain M, Chaudhury P, Vrochides D, Neville A, Cesari M, et al. Pathological response grade of colorectal liver metastases treated with neoadjuvant chemotherapy. HPB 2010; 12: 277–84. doi: 10.1111/j.1477-2574.2010.00170.x 20590898 PMC2873651

[9] Nordlinger B, Sorbye H, Glimelius B, Poston GJ, Schlag PM, Rougier P, et al. Perioperative chemotherapy with FOLFOX4 and surgery versus surgery alone for resectable liver metastases from colorectal cancer (EORTC intergroup trial 40983): a randomised controlled trial. Lancet 2008; 371: 1007–16. doi: 10.1016/S0140-6736(08)60455-9 18358928 PMC2277487

[10] Hoad CL, Palaniyappan N, Kaye P, Chernova Y, James MW, Costigan C, et al. A study of T₁ relaxation time as a measure of liver fibrosis and the influence of confounding histological factors. NMR Biomed 2015; 28: 706–14. doi: 10.1002/nbm.3299 25908098

[11] Banerjee R, Pavlides M, Tunnicliffe EM, Piechnik SK, Sarania N, Philips R, et al. Multiparametric magnetic resonance for the non-invasive diagnosis of liver disease. J Hepatol 2014; 60: 69–77. doi: 10.1016/j.jhep.2013.09.002 24036007 PMC3865797

[12] McDonald N, Eddowes PJ, Hodson J, Semple SIK, Davies NP, Kelly CJ, et al. Multiparametric magnetic resonance imaging for quantitation of liver disease: a two-centre cross-sectional observational study. Sci Rep 2018; ; 8: 9189 15. doi: 10.1038/s41598-018-27560-5 29907829 PMC6003924

[13] Pavlides M, Banerjee R, Sellwood J, Kelly CJ, Robson MD, Booth JC, et al. Multiparametric magnetic resonance imaging predicts clinical outcomes in patients with chronic liver disease. J Hepatol 2016; 64: 308–15. doi: 10.1016/j.jhep.2015.10.009 26471505 PMC4751288

[14] Mole DJ, Fallowfield JA, Welsh F, Sherif A, Kendall TJ, Semple SK.;in press Quantitative magnetic resonance imaging predicts individual future liver performance after liver resection for cancer. PLoS ONE. 10.1371/journal.pone.0238568 PMC7710097 33264327

[15] Hutton C, Gyngell ML, Milanesi M, Bagur A, Brady M. Validation of a standardized MRI method for liver fat and T2* quantification. PLoS One 2018; 13: e0204175. doi: 10.1371/journal.pone.0204175 30235288 PMC6147490

[16] Murakami Y, Abe T, Hashiguchi A, Yamaguchi M, Saito A, Sakamoto M. Color correction for automatic fibrosis quantification in liver biopsy specimens. J Pathol Inform 2013; 4: 36. cited 2020 Nov 26. doi: 10.4103/2153-3539.124009 24524002 PMC3908497

[17] Mole DJ, Fallowfield JA, Kendall TJ, Welsh F, Semple SI, Bachtiar V, et al. Study protocol: HepaT1ca - an observational clinical cohort study to quantify liver health in surgical candidates for liver malignancies. BMC Cancer 2018; 18: 890. doi: 10.1186/s12885-018-4737-3 30208871 PMC6136162

[18] Kleiner DE, Brunt EM, Van Natta M, Behling C, Contos MJ, Cummings OW, et al. Design and validation of a histological scoring system for nonalcoholic fatty liver disease. Hepatology 2005; 41: 1313–21. doi: 10.1002/hep.20701 15915461

[19] Rahbari NN, Garden OJ, Padbury R, Brooke-Smith M, Crawford M, Adam R, et al. Posthepatectomy liver failure: a definition and grading by the International Study group of liver surgery (ISGLS. Surgery 2011; 149: 713–24. doi: 10.1016/j.surg.2010.10.001 21236455

[20] Mojtahed A, Kelly CJ, Herlihy AH, Kin S, Wilman HR, McKay A, et al. Reference range of liver corrected T1 values in a population at low risk for fatty liver disease—a UK Biobank sub-study, with an appendix of interesting cases. Abdom Radiol 2019; 44: 72–84. doi: 10.1007/s00261-018-1701-2 PMC6348264 30032383

